# Esophageal Achalasia: Pros and Cons of the Treatment Options

**DOI:** 10.1007/s00268-022-06495-z

**Published:** 2022-03-03

**Authors:** Mario Costantini, Renato Salvador, Andrea Costantini

**Affiliations:** Department of Surgical, Oncological and Gastroenterological Sciences, Chirurgia Generale 1, University of Padua, 2, via Giustiniani, 35128 Padua, Italy

## Abstract

Achalasia is a primary esophageal motility disorder of unknown origin. The goal of treatment is to reduce the resistance caused by a lower esophageal sphincter that fails to relax and is frequently hypertensive. Many treatment options are available to achieve this goal. In this review, we discuss the pros and cons of each therapeutic approach.

## Introduction

Achalasia is a rare disease, with an incidence of 0.5–1.0 patient per 100,000 population a year [1]. It is nonetheless the most common, and most extensively studied esophageal motility disorder. Despite improvements in our understanding of the pathogenesis of the disease, its etiology remains largely obscure. The appearance of a functional obstruction at the esophagogastric junction (EGJ), and the gradual loss of esophageal peristalsis is responsible for the clinical symptoms, with dilation of the gullet progressing in the final stages to decompensated megaesophagus [1].

As the disease’s etiology is unknown, the aim of current treatments is to deal with the functional abnormalities it causes. There is, unfortunately, no treatment available at present for the loss of peristalsis, so the aim of all therapies in use today is to overcome the functional obstruction of the unrelaxing lower esophageal sphincter (LES), trusting that gravity will then ensure the passage of food through an adynamic gullet. Since pharmacological options for achalasia are impractical, or produce disappointing results, it is treated by paralyzing, tearing or cutting the unrelaxing muscle fibers of the LES, either endoscopically or surgically.

The endoscopic treatment of achalasia with pneumatic dilations (PD) or botulinum toxin (BoT) injections was the mainstay of therapy for the disease for most of the second half of the last century, challenged only in the ’90s by laparoscopic Heller myotomy (LHM) [2]. Endoscopic treatments have lately been revitalized by the introduction and rapid diffusion of per-oral endoscopic myotomy (POEM) [3]. In this paper we review these techniques, pinpointing the pros and cons of each (summarized in Table 1), and suggest a possible flowchart for the treatment of esophageal achalasia in its various manifestations.

**Table 1:** Pros and cons of different treatments for achalasia

Procedure	PROs	CONs
**Botox injection**	Easy to perform	Needs repetition after some months
	Widely available	Gradual loss of efficacy and shortening of symptom-free intervals with further treatments
	No need for general anesthesia (outpatient procedure under sedation)	May interfere with further treatments (myotomy)
	Very low complication rate	
	Ideal for patients unfit for more invasive treatments	
**Pneumatic dilations**	Well standardized protocol	Need to be repeated in about 25% of cases to achieve good long-term results (graded dilations)
	Widely available	Less effective in patients < 40 yrs old
	No need for general anesthesia (outpatient procedure under sedation)	Ineffective in type III achalasia
	Relatively safe (< 5% risk of perforations, usually managed conservatively)	Post-procedural GERD in up to 30% of cases
	No interference with further treatment (LHM or POEM)	
	First choice for treatment after previous myotomy has failed	
**Laparoscopic Heller myotomy**	Relatively safe	Requires general anesthesia
	Excellent short- & mid-term results (> 85%)	Post-op reflux in 10–20% of patients
	Good long-term results (80% at 20 yrs)	Needs a skilled surgeon (learning curve > 20 cases)
	Feasible for all achalasia subtypes and stages	5 small scars
	Feasible for treatment of failures of previous myotomy	Difficult operation when treating failures of previous myotomy
**POEM**	Relatively safe	Requires general anesthesia
	Excellent short- & mid-term results (> 85%)	Need for a skilled endoscopist (learning curve > 50 cases)
	Feasible for all achalasia subtypes and stages (probably best indication is for type III)	Post-op reflux in up to 50% of patients
	Feasible as treatment of failures of previous myotomy	Unknown long-term results
	No scars	

## Botulinum toxin (BoT) injections

This is a sort of “mixed” therapy, in that it implies the use of a pharmacological agent (botulinum toxin A) injected endoscopically into the LES fibers. BoT interferes with neural transmission by blocking the release of acetylcholine at the neuromuscular junction, causing muscle paralysis. This action can thus reduce the LES’s tone, and thereby lower its resistance to the passage of food or saliva [4]. This method was used extensively in the ‘90s because: it is safer and easier than other endoscopic techniques, such as PD; and it does not involve a steep learning curve, so it can also be performed at non-specialist centers. The treatment’s efficacy in lowering LES pressure has been demonstrated in several studies. In general, about 80% of patients show an initial response, but this effect wears off rather quickly in most patients, and a more sustained effect (beyond 6 months) is seen in less than half of the patients treated with BoT injections. A comprehensive meta-analysis showed a symptomatic response rate of 70% at 3 months, 53.3% at 6 months, and 40.6% at 12 months after treatment [5]. Further injections are less effective, probably due to antibody production against the foreign proteins injected [5]. This short-lived efficacy, compared with other treatment options, is the main drawback of this method, which should nowadays be reserved for patients unfit for surgery or invasive endoscopic treatments (PD, POEM). There is also the issue of post-treatment gastro-esophageal reflux, the incidence of which has not been addressed directly, but a symptom-based analysis suggested that it is as high as 20% [4].

## Pneumatic dilation (PD)

Several types of device were developed and used around the middle of the last century to forcefully disrupt an unrelaxing LES. Today, the Rigiflex balloon system (Boston Scientific, Marlborough, MA, USA) is almost always used, making the procedure easier, and leading to some degree of standardization in the use of PD at different centers. The method involves using noncompliant polyethylene balloons, available in three sizes (30, 35, and 40 mm in diameter), mounted on a flexible catheter placed over a guide wire previously inserted endoscopically in the esophagus and across the cardia. Under fluoroscopic control or direct endoscopic guidance, the balloon is gradually inflated with air up to a fixed pressure, which is maintained for a set amount of time. The most important aspects of PD concern the operator’s expertise and the availability of a dedicated surgical backup in the event of perforation requiring surgery. The advantage of PD lies in that it is usually done in an outpatient setting: patients remain under observation for 2–6 h; a liquid diet is allowed in the afternoon; and patients can return to their normal activities the next day. Radiological study with a hydrosoluble contrast is not routinely necessary, but mandatory in the event of any persistent pain or other symptoms after the procedure, to rule out a possible perforation [1]. There are two different strategies for performing PD, one involving a single dilation, the other a series of graded dilations. The latter usually involves an initial dilation at 3.0 cm (recommended for most patients), followed by symptomatic and objective assessments with LES pressure measurements after 4–6 weeks. If patients are still symptomatic, the procedure is repeated with the 3.5 cm balloon, and then even the 4.0 cm balloon, if the symptoms did not improve, or if they recur after 4–6 weeks. One cross-sectional study demonstrated that the symptomatic response to a single PD was 62% at 6 months and then 50%, 38% and 28% at 2, 4 and 6 years, respectively, as opposed to 90% at 6 months, and then 82%, 64% and 44% at 2, 4 and 6 years, respectively, after graded PD [6]. The main disadvantage of PD is, therefore, that symptoms recur over 4–6 years in more than one in two patients, although a better long-term remission rate can be achieved with a strategy of repeat dilations on demand [7]. Predictors of a poor clinical response to PD include: age (< 40 years), male sex, a LES pressure greater than 10–15 mmHg, or a 4sIRP > 20 mmHg on high-resolution manometry after treatment [7]. Males under 40 years old may be less responsive to PD because their LES muscles are thicker. The manometric pattern also has an impact on the outcome of PD: patients with a type I-II pattern benefit more than those with type III [8]. With these limitations, PD can generally be offered to patients with achalasia. It can also be used safely after a failed Heller myotomy. It is now the treatment of choice in such cases, although larger-diameter balloons and repeated sessions are often needed, and the response rate may be less favorable [9]. In a large retrospective series, 105 of 1001 patients (10.5%) whose laparoscopic Heller-Dor (LHD) procedure failed were subsequently treated with one or more sessions of PD, improving their symptoms in all but 11 cases, which eventually required reoperation [10].

The major immediate complication of PD is perforation at the lower end of the esophagus, which occur in about 2% of dilations [7]. Small, asymptomatic perforations can usually be managed conservatively, but large perforations with mediastinal contamination must be surgically repaired [7]. Symptoms of gastroesophageal reflux disease (GERD) occur in 15–35% of patients following PD, but they are usually well controlled with proton-pump inhibitors [7].

## Laparoscopic heller myotomy (LHM)

With the minimally invasive surgery revolution in the early ‘90s, a videoendoscopic approach was promptly applied to myotomy of the LES [2]. This rapidly shifted the balance from PD (the preferred treatment until then) to LHM as the treatment of choice for achalasia. These days, an anterior myotomy of both muscle layers of the EGJ is performed, extending it 2–3 cm onto the proximal stomach. Postoperative GERD may occur following myotomy, as the natural anti-reflux barrier of the LES is completely disrupted, so a fundoplication is usually added [2], the preferred option being an anterior partial fundoplication (Dor, 180°), or a posterior one (Toupet, 270°). A skilled, dedicated surgeon is key to ensuring good results because the learning curve for this procedure has been set at > 20 operations [10], and—given the rarity of achalasia – this is not easy to achieve. This may represent a drawback of the technique, together with the need for general anesthesia. Early success rates with LHM have been high, with a mean of 89% after a median of 35 months (range 8–38) [5], but they may decrease over time. That said, one long-term study on a large cohort found that > 80% of patients were likely to be symptom-free at 20 years [10].

As in the case of PD, patients with type II achalasia have better clinical outcomes after LHM than those with type I, and especially those with type III [8]. On the other hand, while there is no difference in outcome between LHM and PD for patients with type I and II achalasia, patients with type III disease respond better to LHM. This is probably because it enables the muscle fibers above the LES to be severed [8]. Moreover, some manometric studies showed that the LES is longer in type III achalasia than in the other two subtypes, and that extending the myotomy both upwards and downwards could ensure excellent results in this type of achalasia too [11].

There are a few complications associated with LHM, the most frequent involving mucosal perforations, which occur in 2.5–7% of patients [5, 10]. Most of them are detected during the procedure, however, and repaired immediately, with no further consequences for the patient apart from a longer hospital stay. GERD is also reported after LHM, even when a fundoplication is added. In a collective review, postoperative GERD was reported in 8.8% of patients after myotomy with fundoplication, and in up to 31.5% after myotomy without fundoplication [5]. In a recent European achalasia trial, the incidence of GERD after LHD at 5-year follow-up was 23%—which is higher, though not to a statistically significant degree, than after PD (15%) [12]. On the other hand, a recent report on a large series of 1001 patients treated with LHM and Dor fundoplication at an experienced center found that only 9.1% had GERD confirmed by 24-h pH-manometry [10].

LHM can also be used as a rescue treatment after a previous myotomy has failed and patients have not responded to complementary PD. A recent report showed that, although this procedure is difficult (the failure rate is higher than after primary LHM, and postoperative GERD is more likely), it is safe and effective in relieving symptoms in such patients with highly refractory achalasia [13].

## Per-oral endoscopic myotomy (POEM)

First described in an animal model by Pasricha et al. in 2007, POEM was optimized and brought to the clinical practice by Inoue et al. in 2010 [3], and its use spread rapidly first in Asia, and then, in the USA and Europe. A meta-analysis reported on 2,373 patients treated with POEM in 12 countries, two-thirds of them in Japan and China [14]. All reports to date indicate that the technique achieves excellent results (> 90%) in the short term, but it is worth emphasizing that all these enthusiastic data come from uncontrolled open-label studies with a short follow-up. Studies with longer follow-up have become available only recently. Von Renteln et al. [15] found that the success rates decreased over time, from 97% after 3 months to 89% after 6 months, and 82% after 1 year. Werner et al. similarly reported the success rate declining to 79% at 2-year follow-up [16]. As in the case of PD and LHM, the efficacy of POEM thus seems to fade over time.

POEM would appear to be ideal for treating longer-segment disorders of esophageal peristalsis like type III achalasia, or diffuse esophageal spasm. Studies on POEM used specifically for type III achalasia showed that it is safe and effective, achieving long-term symptom relief in more than 90% of cases [17]. POEM could therefore represent the first-line approach to such patients.

On the whole, POEM seems to be a safe technique. A recent study on 1,826 patients reported adverse events in an overall 7.5% of cases, most of them mild (6.4%, while 1.7% were moderate, and only 0.5% were severe [18]. A recent meta-analysis on 22 studies identified only one death out of 1,122 patients (a mortality rate of less than 0.1%) [19].

GERD is the main drawback of POEM reported to date, since no anti-reflux procedure is associated with the myotomy. Based on objective observations, the number of patients developing GERD after POEM reportedly ranges from 10 to 57% [19, 20]. Some cases of new-onset Barrett’s esophagus, and one of peptic stricture have also been reported less than 2 years after the procedure [16]. Most importantly, a recently published case of adenocarcinoma of the esophagus following POEM casts a worrisome shadow over this procedure [21]. Another concern lies in that the technique involved in POEM demands considerable endoscopic skills. Initial studies on the learning curve for POEM suggested that more than 50 cases needed to be treated to reduce the risk of failures and adverse events. This raises the question again of how such quotas can be reached by the average endoscopist when achalasia is such a rare disease [22]. The learning curve may be as low as 15–25 cases, however, for endoscopists with plenty of previous experience of advanced therapeutic interventions [16]. Be that as it may, seeing the ease with which POEM has been introduced in the daily clinical practice of many endoscopists—even though its long-term efficacy and related complications, such as GERD and fibrosis, have yet to be assessed—gives us cause for concern.

POEM has also been used to treat patients after LHM has failed. A recent meta-analysis on 9 retrospective studies from specialized high-volume centers reported satisfactory results in 90% of 270 patients treated, with adverse events in only 10.8% of cases (most of them minor, involving conservatively managed mucosal perforations) [23]. POEM is particularly attractive in this context because it enables an extended myotomy in a new plane (different from the anterior one) to be completed more easily and with fewer risks than a laparoscopic revision. POEM after LHM comes with the additional benefit that most patients will have had an anti-reflux procedure as part of their first operation, and this minimizes the risk of GERD. Even in patients who have already had a POEM, it has subsequently proved technically feasible to perform a new POEM in a new plane, albeit with somewhat lower clinical success rates (around 85%), and slightly higher complication rates (around 17%) [16]. More robust evidence is needed, however, before this approach can be wholeheartedly recommended.

## Direct comparison between treatments

### Botox injections versus pneumatic dilatations and/or laparoscopic Heller myotomy

BoT injection therapy has been compared with both PD and surgical myotomy. A meta-analysis of controlled studies comparing BoT injections with PD showed that the latter obtained better results (the response rate at 1 year was 66% with PD versus 36% with BoT injections (p < 0.0001) [4]. In the only multicenter RCT comparing BoT injections with LHM [24], the latter yielded better response rates at 1 year (83% versus 65% with BoT, *p* = 0.03). The probability of being symptom-free at 2 years was 87.5% after surgery, but only 34% after BoT (p < 0.05). In the light of these findings, BoT treatment is now rightly reserved for high-risk patients too ill to undergo more invasive therapies (PD, POEM or LHM), or in whom any complications of such therapies (perforation) cannot be treated surgically.

### Pneumatic dilation versus laparoscopic Heller myotomy

Until recently, the choice between PD and LHM as the most appropriate treatment for a given patient was based mainly on the locally available endoscopic or surgical expertise. In a review of case series from 1989 to 2006, Campos et al. reported an overall 68% symptom improvement rate in 1,065 patients undergoing PD as compared with an 89% improvement rate in 3,086 patients treated with LHM [5]. The results of an RCT comparing PD with LHM plus Dor’s fundoplication (LHD) were published in 2011 [12]. This was a European Achalasia Trial in which patients from five European countries were randomly assigned to graded PD (n = 94 patients; 30- and 35-mm balloons and up to 3 repeat dilations) or LHD (n = 106). The two treatments were comparable in terms of symptom relief at 2 years: 86% for PD, 90% for LHD. When the 5-year follow-up results were published [25], there was still no significant difference in the success rates of the two treatments (82% after PD, and 84% after LHD, *p* = 0.92), but 25% of the patients treated with PD had needed further dilations in the meantime. Age < 40 years, prior daily chest pain, esophageal diameter < 4 cm before treatment, and type III achalasia were identified as predictors of PD failing and repeat dilation being needed, whereas none of these factors predicted the failure of LHD. The results of this study contribute important, scientifically sound information on the treatment of esophageal achalasia, showing that PD can still be considered as effective as LHM for the treatment of this rare, but often cumbersome disease.

### Pneumatic dilatation versus POEM

PD has also been retrospectively compared with POEM. In a recently published meta-analysis of 7 studies [26], a good outcome was reported at 12 and 24 months in 94.9% and 91.7% of 298 patients treated with POEM, and in 74.9% and 63.8% of 321 patients treated with PD (*p* < 0.001). The rates of complications and GERD were higher in the POEM group. No information was provided on the PD protocol used. The results of a multicenter RCT comparing POEM with PD, performed in 67 and 66 patients, respectively, were published in 2019 [27]. At 2-year follow-up, 92% of the patients in the POEM group had good results, as opposed to 54% of those in the PD group (*p* < 0.01). GERD was reported in 41% of the patients treated with POEM and in only 7% of those managed with PD. This important study is flawed by the PD protocol used, which envisaged only 1–2 graded dilations (starting with a 3.0 cm balloon, with a repeat dilation with a 3.5 cm balloon a few weeks later if the symptom response was inadequate). Had the 14 patients subsequently treated with an additional dilation procedure been included, the success rate for PD would have been 76%. As mentioned earlier, several studies have shown that an “on demand” approach to PD achieves far better results than a single dilation session. In clinical practice, patients have repeat dilations if their symptoms recur, whereas such situations were considered as treatment failures in the RCT.

### Laparoscopic Heller myotomy versus POEM

Only a few years after its introduction in 2010, and its rapid and widespread diffusion, POEM was compared with LHM in several studies and meta-analyses. All these studies found the results achieved with the two methods comparable, or better for POEM, at short- or medium-term follow-up. For example, Schlottmann et al. [28] found the predicted probabilities of improvement in dysphagia were 93.5% for POEM, and 91.0% for LHM at 12 months (*p* < 0.01), and 92.7% for POEM, and 90.0% for LHM at 24 months (*p* < 0.01). The follow-up for the two methods differed, however, being significantly longer for studies on LHM than for those on POEM (41.5 vs. 16.2 months, respectively, *p* < 0.0001). Patients had more GERD after POEM, as demonstrated by the erosive esophagitis identified in 11.5% of patients treated with LHM, and 22.4% of those treated with POEM (*p* < 0.0001), or by pH monitoring (in 11.1% and 47.5% of patients, respectively; *p* < 0.0001). A recent comparative study using propensity scores for 140 patients treated at a center of excellence for LHM, and 140 treated at a center of excellence for POEM during the same time span showed similar outcomes after a median 31 and 24 months of follow-up, respectively: the treatments were successful in 95.7% of the former group and 99.3% of the latter (*p* = 0.12), and both groups had much the same low rate of complications [29]. A sizable proportion of the patients in both groups underwent function studies 6 months after their myotomy, which revealed a higher percentage of pH-proven reflux in the POEM group than in the LHM group (38.4% vs. 17.7%; *p* < 0.001). The same was true of cases of endoscopically confirmed esophagitis (37.4% vs. 15.2%, respectively; *p* < 0.001). It should be noted, however, that the surgical procedure for the LHM group has always included a Dor fundoplication, as was customary at the center involved.

Finally, the results of a multicenter RCT involving eight European centers were published in 2019 [30]. There were 112 patients randomized to POEM and 109 to LHM plus a Dor fundoplication (LHD). The clinical success rate at 2-year follow-up was 83.0% for the POEM group, and 81.7% for the LHD group, with an absolute between-group difference of 1.4% in favor of POEM (*p* = 0.007 for noninferiority). This study confirmed a higher incidence of post-procedural reflux esophagitis in the POEM group than in the LHD group, both at 3 months (57% vs. 20%) and at 24 months (44% vs. 29%). The POEM group also had a higher proportion of patients with abnormal acid exposure at 3-month follow-up (44% vs. 33% in the LHD group), though this difference did not reach statistical significance, and disappeared at 2-year follow-up (30% in both groups). There were more patients in the POEM group than in the LHD group taking low-dose proton-pump inhibitors, however, both at 3-month (30.6% vs. 27.6%) and at 24-month follow-up (52.8% vs. 27.2%).

## Conclusions

From the above analysis of the pros and cons of the techniques available for treating achalasia, it seems they are all appropriate—apart from BoT injections, which should be reserved for patients too ill to undergo more invasive options.

Patients with achalasia can be offered PD, LHM (with fundoplication) or POEM, after illustrating the merits and drawbacks of the different options (Table 2). Generally speaking, the choice of treatment should be left to the patient, not based on the expertise or preferences of their doctor. PD is certainly still an attractive option as it is now well standardized, widely available, relatively safe, and does not require general anesthesia. But it has its limitations, and is generally less effective than LHM or POEM, especially in certain circumstances. It therefore should not be offered to children and young adults (< 40 years old), or to patients with type III achalasia. On the other hand, complementary PD may be the treatment of choice after a failed LHM or POEM, as it usually relieves residual dysphagia in most patients.

**Table 2:** Suggested indications for different treatments in different disease-related and patient-related phenotypes of achalasia

	**Botox**	**PD**	**LHD**	**POEM**
Achalasia in general	No	Yes	Yes	Yes
Achalasia in patients < 40 years old	No	No	Yes	Yes (?)
End-stage achalasia	No	No	Yes	Yes
Type III achalasia	No	No	Yes	Yes
Children and teenagers	No	No	Yes	No
Achalasia recurrence after LHM	No	Yes (1^st^ choice)	Yes (3^rd^ choice)	Yes (2^nd^ choice)
Poor candidates for surgery	Yes	No	No	No

LHM and POEM have a similar profile in terms of efficacy, perioperative complications, and adverse events. It is worth emphasizing, however, that POEM is an invasive procedure (despite being managed endoscopically) that requires general anesthesia, like LHM. Both techniques demand skilled surgeons and endoscopists, and should only be performed at high-volume centers to ensure the best overall results.

The rate of GERD after POEM (always reportedly higher than after LHM with fundoplication) should deter doctors from offering POEM as a first-line therapy in younger people (and especially children) with a long life expectancy. More data on the long-term effects of such iatrogenic reflux are obviously needed before any definitive conclusions can be drawn on the issue. Even if post-POEM GERD does not lead to relevant complications, such as severe esophagitis, Barrett’s esophagus or adenocarcinoma of the cardia, it will certainly require long-term PPI use and dietary restrictions for one in two patients. POEM is still most indicated for the treatment of type III achalasia, or after LHM has failed in patients unresponsive to PD.

In conclusion, the choice of treatment for achalasia is multifactorial, and a “one size fits all” approach is not appropriate. All treatments and all patients have various aspects that need to be examined thoroughly before opting for one treatment rather than another. A center specializing in achalasia should have all possible techniques in its armamentarium so that it can choose the most appropriate treatment for a given patient.
